# Forensic Efficiency Estimation of a Homemade Six-Color Fluorescence Multiplex Panel and In-Depth Anatomy of the Population Genetic Architecture in Two Tibetan Groups

**DOI:** 10.3389/fgene.2022.880346

**Published:** 2022-05-27

**Authors:** Yanfang Liu, Wei Cui, Xiaoye Jin, Kang Wang, Shuyan Mei, Xingkai Zheng, Bofeng Zhu

**Affiliations:** ^1^ Guangzhou Key Laboratory of Forensic Multi-Omics for Precision Identification, School of Forensic Medicine, Southern Medical University, Guangzhou, China; ^2^ Laboratory of Fundamental Nursing Research, School of Nursing, Guangdong Medical University, Dongguan, China; ^3^ Department of Forensic Medicine, Guizhou Medical University, Guiyang, China; ^4^ Ningbo Health Gene Technologies Co., Ltd., Ningbo, China; ^5^ Key Laboratory of Shaanxi Province for Craniofacial Precision Medicine Research, College of Stomatology, Xi’an Jiaotong University, Xi’an, China; ^6^ Microbiome Medicine Center, Department of Laboratory Medicine, Zhujiang Hospital, Southern Medical University, Guangzhou, China

**Keywords:** forensic efficiency estimation, population genetics, Tibetan group, genetic architecture dissection, deletion/insertion polymorphisms

## Abstract

The genetic information of the Chinese Tibetan group has been a long-standing research hotspot among population geneticists and archaeologists. Herein, 309 unrelated individuals from two Tibetan groups living in Qinghai Province, China (CTQ), and Tibet Autonomous Region, China (CTT), were successfully genotyped using a new homemade six-color fluorescence multiplex panel, which contained 59 autosomal deletion/insertion polymorphisms (au-DIPs), two mini short tandem repeats (miniSTRs), two Y-chromosomal DIPs, and one Amelogenin. The cumulative probability of matching and combined power of exclusion values for this new panel in CTQ and CTT groups were 1.9253E-27 and 0.99999729, as well as 1.5061E-26 and 0.99999895, respectively. Subsequently, comprehensive population genetic analyses of Tibetan groups and reference populations were carried out based on the 59 au-DIPs. The multitudinous statistical analysis results supported that Tibetan groups have close genetic affinities with East Asian populations. These findings showed that this homemade system would be a powerful tool for forensic individual identification and paternity testing in Chinese Tibetan groups and give us an important insight for further perfecting the genetic landscape of Tibetan groups.

## 1 Introduction

Critical samples from crime scenes may contain only small amounts of highly fragmented and damaged DNA, so forensic scientists make every effort to address this complex problem. Numerous research approaches have emerged to amplify shorter amplicons of the target sequences in the damaged DNA samples from mass disasters or forensic caseworks (Senge et al., 2011). Short tandem repeats (STRs) have been authenticated to be highly sensitive, dependable, and discriminating for parentage testing and personal identification (Gymrek, 2017). In consideration of the same characteristics as the STRs, miniSTR typing is the representative analytical method of first choice for the degraded biological samples (Graham, 2005). More accurate DNA profiles can be achieved through miniSTRs, whose primers are located more closely to the repeat regions of STRs, so as to improve genotyping success ratio (Kun, Wictum, and Penedo, 2018). Currently, a series of studies have also suggested that single nucleotide polymorphism (SNP) are the efficacious genetic markers for forensic identification of degradation samples, but there is limited prevalence of SNP kits in forensic laboratories due to their high cost and tedious detection procedures based on the capillary electrophoresis analysis or other platforms (Fondevila et al., 2012). Deletion/insertion polymorphisms (DIPs) with smaller amplicon sizes and a time-saving genotyping process have been widely favored in the forensic molecular biology field (Fondevila et al., 2012). In our prophase research, we constructed a new homemade six-color fluorescence multiplex PCR system encompassing one Amelogenin gene, two Y-chromosome DIPs (Y-DIPs), 59 highly polymorphic autosomal DIPs (au-DIPs), and two mini short tandem repeats (miniSTRs) with amplicon lengths within 200 bp, and it was devoted to detect degradation samples for personal identification and paternity testing in the East Asian region (Liu et al., 2022). However, the genetic distributions and forensic applicability of these genetic markers in the novel panel have not yet been investigated and studied in most populations from China. The genetic polymorphisms of these genetic markers and forensic application efficiency estimation for Chinese Tibetan groups in different regions are important contents in this study.

Tibetan group is one of the nationalities with historic backgrounds, diligence, courage, and wisdom in China. The regions where Tibetans live are near the Tibetan Plateau, which is inlaid with snow-capped highlands and mountains for most of the year, resulting in a relatively geographical closure. Their pronounced historical traditions, distinctive culture, and genetic characteristics made them a long-term focus to explore hotspots among population geneticists and archeologists. In recent decades, many scholars’ studies on archeology (Aldenderfer, 2011), language (Sagart et al., 2019), clothing (Li, Guo, and Wang, 2012), Tibetan medicine (Dakpa, 2014), genetics (Hu et al., 2017; Fan et al., 2021), and various aspects have remarkably enhanced our knowledge of Tibetan ethnicity. Nevertheless, these studies, other than the DNA studies, which veritably reflect human evolution, are susceptible to external factors such as the environment or subjective factors. In a previous study, genetic affinities among Tibetan groups and other worldwide populations were explored using 35 DIPs (Liu et al., 2020). But the currently available limited genetic information of Tibetan groups have also been made this topic still need to be explored sufficiently.

Thus, the present research intends to estimate the effectiveness of the new homemade six-color fluorescence multiplex system in Tibetan groups and further perfect the genetic landscape of the Tibetan groups from the perspective of the 59 au-DIPs. This study will be a significant contribution toward understanding the genetic background and diversity of Tibetan group.

## 2 Materials and Methods

### 2.1 Characteristics of the Loci in the New Panel

The genetic markers have been carefully selected and constructed in a new homemade six-color fluorescence multiplex panel (Liu et al., 2022). The 2 Y-DIPs and 2 miniSTRs in the panel were selected from previously used loci. Physical distances among genetic markers located on autosomes are greater than 10 Mb. The 59 au-DIPs are located in intronic regions with allele lengths of 2–10 bp and the minor allele frequencies are greater than 0.2; and the *F*-statistics (*F*_ST_) values for these loci among the five East Asian populations from the 1000 Genomes Project database were less than 0.06.

### 2.2 Sample Collection, PCR Amplification, and Genotyping

Bloodstain specimens of unrelated healthy individuals examined in the present study were gathered from the Tibetan group in Qinghai Province, China (CTQ), and the Tibetan group in the Tibet Autonomous Region, China (CTT), with the sample sizes of 155 and 154, respectively. The present research was conducted according to the ethical guidelines of the Southern Medical University and Xi’an Jiaotong University Health Science Center and further authorized by the Ethical Committee of the Xi’an Jiaotong University Health Science Center (approval number: 2019-1039).

Bloodstain samples were amplified via the GeneAmp PCR system 9700 (Thermo Fisher Scientific, Waltham, United States). About 1.0 mm^2^ was used as template in a reaction mix with 10 µl total volume, comprising 2 µl of Master Mix (HEALTH Gene Technologies, Ningbo, China), 1 µl of Primer Mix, and 7 µl of nuclease-free water. The cycling conditions were as follows: an initial denaturation step of 5 min at 95°C; followed by 2 cycles of 94°C for 10 s and annealing at 63°C for 90 s; and then 23 cycles of 94°C for 10 s, annealing at 60°C for 90 s, and extension at 60°C for 90 s; and then final extension at 60°C for 15 min. Then, 1.0 µl PCR product was mixed with 0.5 µl of an internal lane size standard and 8.5 µl of Hi-Di deionized formamide. The mixtures were denatured at 95°C for 3 min, cooled immediately for 3 min, and then genotyped on the Applied Biosystems^®^ 3500xL Genetic Analyzer (Thermo Fisher Scientific) for electrophoretic separation and detection. Finally, GeneMapper ID version 3.2 software (Thermo Fisher Scientific) was utilized to determine the genotyping results.

### 2.3 Comparison Populations and Data Analyses

The 26 comparison population data from five different geographic regions (Africa, Europe, America, East Asia, and South Asia) were downloaded from the 1000 genomes database. The statistical analysis information used in this study is summarized in Table 1.

**TABLE 1:** Statistical analysis information for forensic parameters and population genetic analyses.

Statistical parameter	Software	Description
Exact tests of Hardy–Weinberg equilibrium (HWE-*p*)	STRAF tool (Gouy and Zieger, 2017)	Sample representativeness, locus independence testing, genetic polymorphism, and forensic parameter analysis
Linkage disequilibrium (LD) analysis	-	-
Allele frequency	-	-
Power of exclusion (PE)	-	-
Power of discrimination (PD)	-	-
Probability of matching (PM)	-	-
Polymorphic information content (PIC)	-	-
Observed heterozygosity (H_obs_)	-	-
Allele frequency heatmap	TBtools version 0.665 (Chen et al., 2020)	Insertion allele frequency distribution characteristics of the 59 au-DIPs in the CTQ and CTT groups, and the other 26 reference populations
*F*_ST_ genetic distance	Arlequin version 3.5 (Excoffier and Lischer, 2010)	Population differentiation among the two studied Tibetan groups and other 26 reference populations
*Nei’*s genetic distance (*D*_A_ distance)	DISPAN program	*D*_A_ distances among the two studied Tibetan groups and the other 26 reference populations, formed under the assumption that genetic differences originated from genetic drift and mutation events (Jin et al., 2019)
Phylogenetic tree reconstructions	MEGA version 7.0 (Kumar, Stecher, and Tamura, 2016)	Rooted evolutionary tree, which was built based on the *F*_ST_ values among the pairwise populations by the unweighted pair group method with the arithmetic mean (UPGMA) method
	Phylip version 3.697 (Shimada and Nishida, 2017)	Unrooted evolutionary tree, namely, radiation tree, which was established based on the allelic frequency data using the neighbor-joining method
Principal component analyses (PCA)	*R* Studio	Population level PCA based on the allele frequencies of the same loci
	Origin 2021	Individual level PCA based on the allelic genotyping raw data
	*R* version 3.5.3	Contribution quality correlation circle of the locus in the corresponding PCA plots
Population genetic structure analyses	STRUCTURE version 2.3.4 (Porras-Hurtado et al., 2013)	Each *K* value (*K* = 2-7) with ten replicates run under the admixture model includes 10,000 burn-in period length, followed by 10,000 Markov chain Monte Carlo steps
	CLUMPP version 1.1.2 (Jakobsson and Rosenberg, 2007)	The ancestor component bar graph drawing after the population genetic structure analysis
	Distruct version 1.1 (Rosenberg, 2004)	-
	Structure Harvester program (Earl and vonHoldt, 2012)	The optimum *K* value determination of STRUCTURE analysis
Population-specific divergence (PSD)	Snipper version 2.5 http://mathgene.usc.es/snipper/	The accumulated PSD values of all loci in distinguishing different geographical region populations
Informativeness for assignment (*I*_n_) and *F*_ST_ values of locus-by-locus AMOVA	Infocalc version 1.1 https://rosenberglab.stanford.edu/infocalc.html	Determining the level of information about individual ancestry provided by each locus, and the locus-by-locus AMOVA *F*_ST_ values of each locus to pairs of regional populations

## 3 Results

### 3.1 Forensic Genetic Parameter Analyses of the New Panel

**FIGURE 1:**
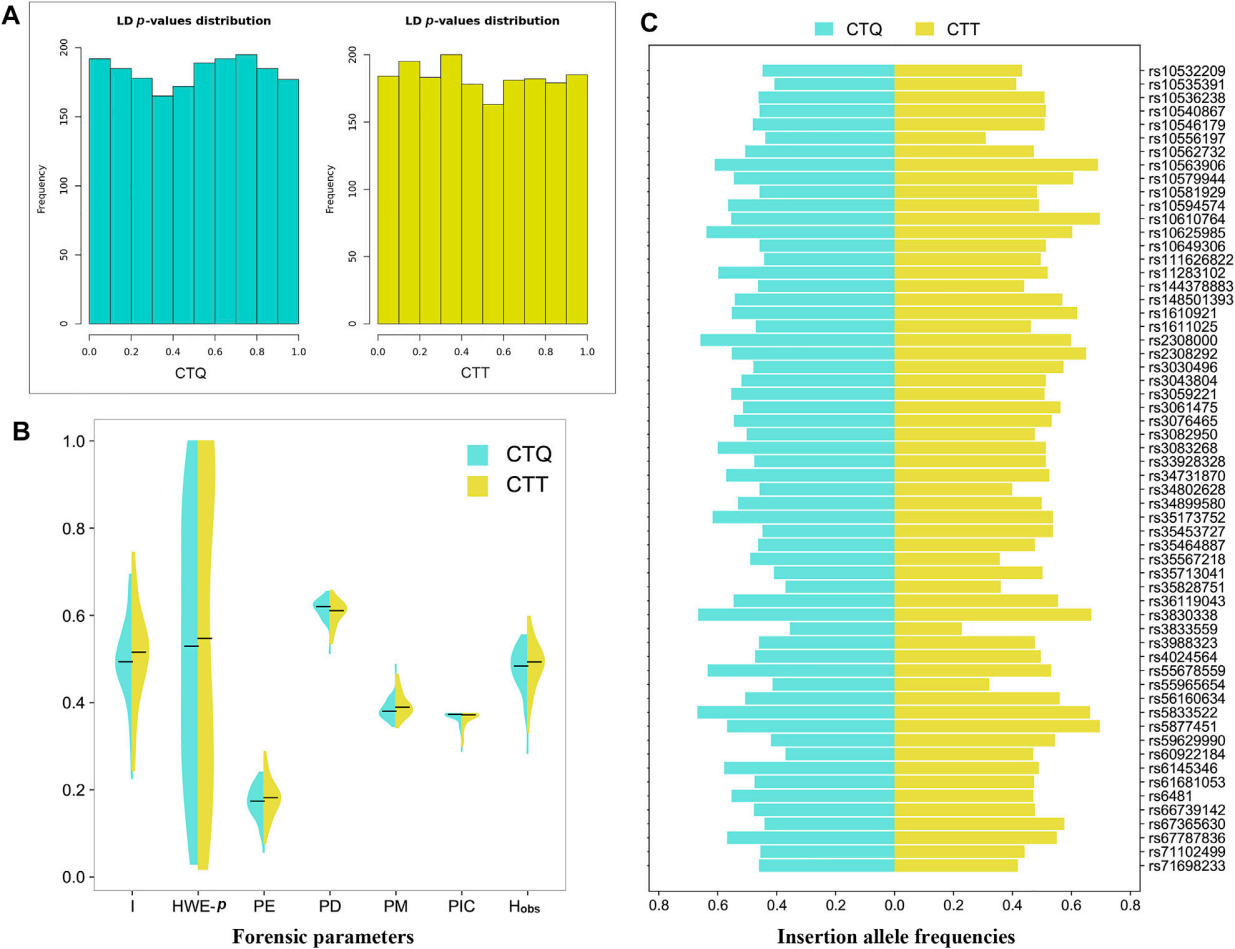
Forensic parameters in the CTQ and CTT groups. **(A)** Bar chart of *p*-value distributions of pairwise linkage disequilibrium tests at the 59 au-DIPs and two miniSTRs. **(B)** Half violin of some forensic parameters for the 59 au-DIPs and two miniSTRs. I, insertion allele frequencies; HWE-*p*, exact tests of Hardy–Weinberg equilibrium; PE, power of exclusion; PD, power of discrimination; PM, probability of matching; PIC, polymorphic information content; H_obs_, observed heterozygosity. **(C)** Half horizontal bar of insertion allele frequencies at the 59 au-DIPs.

For the two studied Tibetan groups, *p* values of the LDs (Figure 1A) for all loci combinations of 59 au-DIPs and two miniSTRs were higher than 0.0003 in the CTQ group (Supplementary Table S1) and higher than 0.0005 in the CTT group (Supplementary Table S2), which signified that no LDs for pairwise loci were discovered in these two groups after applying the Bonferroni correction (*p* > 0.05/1830 = 0.00002732). The *p* values of the HWE exact tests are displayed in Supplementary Table S3 and Figure 1B. For the two studied Tibetan groups, the HWE *p* values ranged from 0.0300 to 1.0000 and from 0.0180 to 1.0000 in the CTQ and CTT groups, respectively. Some slight deviations for HWE were observed at loci rs10581929 (HWE-*p* = 0.0320), rs35828751 (HWE-*p* = 0.0300), rs3830338 (HWE-*p* = 0.0450), and rs3833559 (HWE-*p* = 0.0300) in the CTQ group, and at loci D1S1656 (HWE-*p* = 0.0110), rs3059221 (HWE-*p* = 0.0310), rs66739142 (HWE-*p* = 0.0280), and rs67365630 (HWE-*p* = 0.0180) in the CTT group. After conducting the Bonferroni correction (0.05/61 = 0.0008), there were no deviations from HWE at these loci.

Herein, the allele frequencies and other forensic parameters are summarized in Supplementary Tables S3, S4 and Figures 1B, C. A total of 136 alleles were confirmed at the 59 au-DIPs and the two miniSTRs in the CTQ group. The insertion allele frequencies of 59 au-DIPs ranged from 0.2260 (rs3833559) to 0.6940 (rs5877451), with a median value of 0.4940. The allele frequencies of D1S1656 and D3S1358 loci ranged from 0.0060 to 0.4130. The PE, PD, PM, PIC, and H_obs_ values of 59 au-DIPs ranged from 0.0571 (rs3833559) to 0.2401 (rs10546179 and rs1611025); from 0.5126 (rs3833559) to 0.6547 (rs10581929); from 0.3453 (rs10581929) to 0.4874 (rs3833559); from 0.2885 (rs3833559) to 0.3750 (rs10540867, rs10649306, rs11283102, rs3043804, rs3083268, rs33928328, rs34899580, rs35713041, and rs4024564); and from 0.2839 (rs3833559) to 0.5548 (rs10546179 and rs1611025), with median values of 0.1738, 0.6199, 0.3801, 0.3732, and 0.4839, respectively. The PE, PD, PM, PIC, and H_obs_ values of the two miniSTR loci D1S1656 and D3S1358 were 0.6727, 0.9496, 0.0504, 0.8094, and 0.8387, as well as 0.3942, 0.8650, 0.1350, 0.6556, and 0.6774, respectively. The cumulative PM (CPM), cumulative PD (CPD), and combined PE (CPE) values of the 59 au-DIPs in the CTQ group were 2.8296E-25, 1-2.8296E-25, and 0.9999863, respectively. After adding the two miniSTRs, their CPM, CPD, and CPE values reached to 1.9253E-27, 1-1.9253E-27, and 0.99999729, respectively.

A total of 138 alleles were detected at the 59 au-DIPs and the 2 miniSTRs in the CTT group (Supplementary Tables S3, S4). The insertion allele frequencies of the 59 au-DIPs ranged from 0.2440 (rs10556197) to 0.7440 (rs3830338), with a median value of 0.5160. The allele frequencies of D1S1656 and D3S1358 loci were from 0.0030 to 0.3860. The PE, PD, PM, PIC, and H_obs_ values of the 59 au-DIPs were from 0.0772 (rs10556197) to 0.2878 (rs66739142); from 0.5351 (rs10556197) to 0.6570 (rs3059221); from 0.3430 (rs3059221) to 0.4649 (rs10556197); from 0.3006 (rs10556197) to 0.3750 (rs3061475, rs4024564, and rs61681053); and from 0.3312 (rs10556197) to 0.5974 (rs66739142), with median values of 0.1819, 0.6106, 0.3894, 0.3719, and 0.4935, respectively. The PE, PD, PM, PIC, and H_obs_ values of the D1S1656 and D3S1358 loci were 0.7349, 0.9297, 0.0703, 0.8022, and 0.8701, and 0.5153, 0.8441, 0.1559, 0.6482, and 0.7532, respectively. The CPM, CPD, and CPE values of the 59 au-DIPs in the CTT group were 1.3742E-24, 1-1.3742E-24, and 0.9999919, respectively. After adding the two miniSTR loci, their CPM, CPD, and CPE values reached to 1.5061E-26, 1-1.5061E-26, and 0.99999895, respectively.

### 3.2 Allele Frequency Divergences of the 59 au-DIPs

Supplementary Figure S1 lists the insertion allele frequency values of the 59 au-DIPs in two studied Tibetan groups and 26 reference populations involved in this study and intuitively revealed the allele frequency divergences among these 28 populations. The color gradation from turquoise to yellow and then to purple meant the transition of insertion allele frequency values from the lowest to the highest. Except that the insertion allele frequency values of rs3833559 in the CTQ and CTT groups, and of rs10556197 and rs55965654 in the CTT group were less than 0.3, the insertion allele frequency values of the 59 au-DIPs were relatively balanced in the CTQ and CTT groups, and the other five populations from East Asia with the frequency values ranging from 0.3 to 0.7. From the aspect of heatmap clustering (Supplementary Figure S1), the branch on the left represented the cluster based on the insertion allele frequencies of each locus in these populations, and the branch above indicated the cluster pattern of the 28 populations based on the insertion allele frequencies of the whole 59 DIPs. By and large, two major cluster classifications (African population cluster and non-African population cluster) appeared in all 28 populations; and the loci in the same small branch reflected similar allele frequency distributions in different populations from the same geographic region.

### 3.3 Population Genetic Distance Measures

**FIGURE 2:**
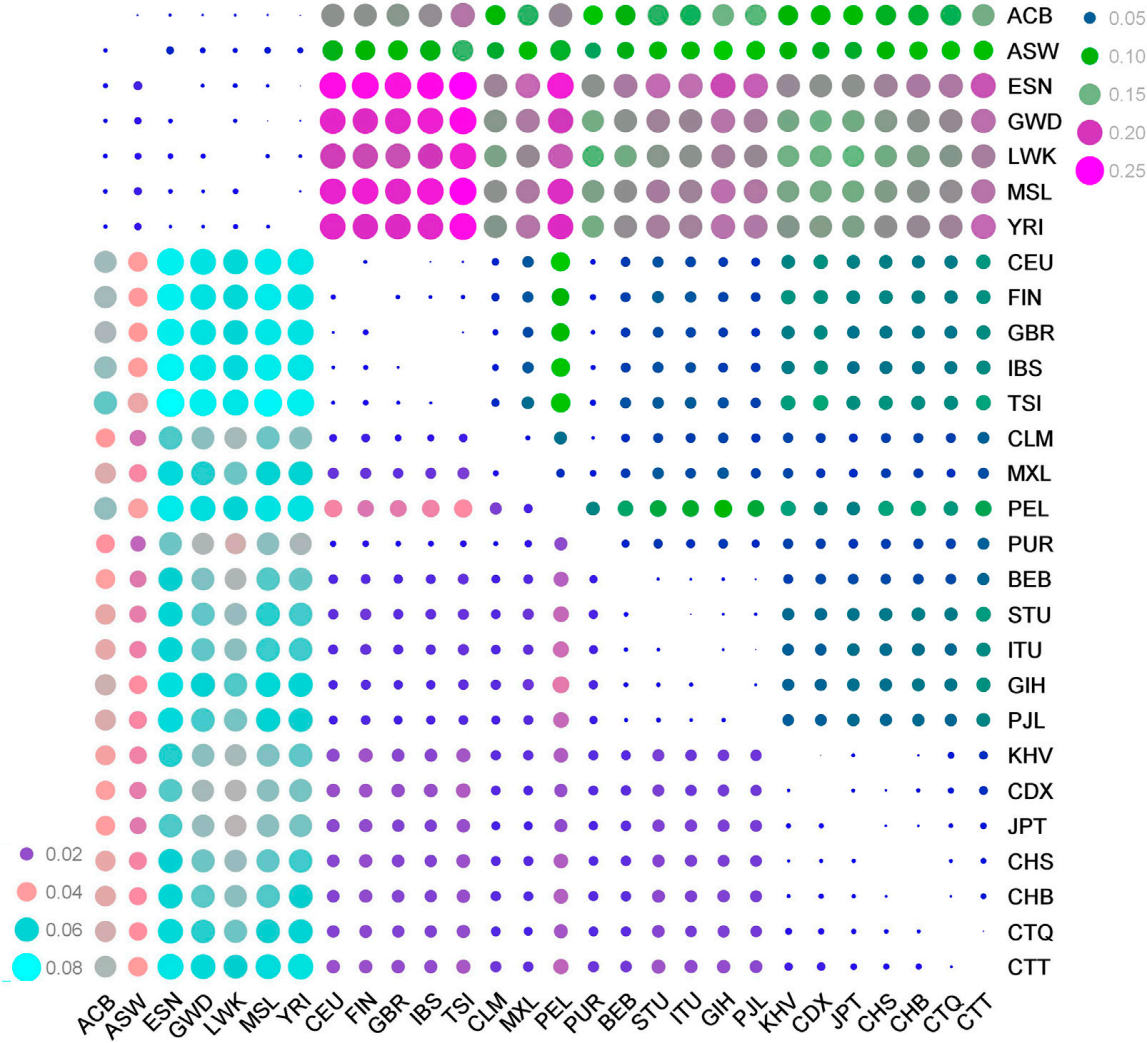
Distribution heatmaps of the pairwise *F*_ST_ values (the upper right part) and *D*_A_ values (the bottom left part) among the CTQ and CTT groups and other 26 comparison populations. CTQ, Tibetan in Qinghai Province, China (n = 155); CTT, Tibetan in Tibet Autonomous Region, China (n = 154); CDX, Chinese Dai in Xishuangbanna, China (n = 93); CHB, Han Chinese in Beijing, China (n = 103); CHS, Southern Han Chinese, China (n = 105); KHV, Kinh in Ho Chi Minh City, Vietnam (n = 99); JPT, Japanese in Tokyo, Japan (n = 104); PJL, Punjabi in Lahore, Pakistan (n = 96); GIH, Gujarati Indian in Houston, TX (n = 103); ITU, Indian Telugu in the United Kingdom (n = 102); STU, Sri Lankan Tamil in the United Kingdom (n = 102); BEB, Bengali in Bangladesh (n = 86); CLM, Colombian in Medellin, Colombia (n = 94); MXL, Mexican Ancestry in Los Angeles, California (n = 64); PEL, Peruvian in Lima, Peru (n = 85); PUR, Puerto Rican in Puerto Rico (n = 104); CEU, Utah residents with Northern and Western European ancestry (n = 99); FIN, Finnish in Finland (n = 99); GBR, British in England and Scotland (n = 91); IBS, Iberian populations in Spain (n = 107); TSI, Toscani in Italy (n = 107); ACB, African Caribbean in Barbados (n = 96); ASW, African Ancestry in Southwest US (n = 61); ESN, Esan in Nigeria (n = 99); GWD, Gambian in Western Division, The Gambia (n = 113); LWK, Luhya in Webuye, Kenya (n = 99); MSL, Mende in Sierra Leone (n = 85); and YRI, Yoruba in Ibadan, Nigeria (n = 108).

The *F*_ST_ values and *D*_A_ distances of pairwise populations based on the 59 au-DIPs were used to perform genetic relationship analyses from these diverse genetic distances. The results showed (Supplementary Tables S5, S6, Figure 2) that the minimal *F*_ST_ value (*F*_ST_ = 0.0016) and *D*_A_ distance (*D*_A_ = 0.0012) were between the CTQ and CTT groups. The second smallest *F*_ST_ value and *D*_A_ distance were between the CTQ group and the CHB population (*F*_ST_ = 0.0035, *D*_A_ = 0.0019) from East Asia, and then between the CTT group and the CHS population (*F*_ST_ = 0.0107, *D*_A_ = 0.0038) from East Asia. The maximum *F*_ST_ values (0.1334 in CTQ, 0.1439 in CTT) and *D*_A_ distances (0.0439 in CTQ, 0.0469 in CTT) were found between the Tibetan groups and Esan in Nigeria (ESN) from Africa. In the heatmaps of *F*_ST_ (the upper right part of the picture) and *D*_A_ distance (the bottom left part of the picture) values (Figure 2), the color degree ranged from purple to pink and then to turquoise, as well as the color degree from blue to green to rose-red denoted the changes from minimum to maximum population hereditary distances. Obviously, pairwise comparisons among the Tibetan groups and the five East Asian populations preferred the small bubbles representing relatively close genetic distances, while the pairwise comparison populations among the Tibetan groups and the African populations were inclined toward the big bubbles illustrating the farther hereditary distances.

### 3.4 Phylogenetic Reconstructions

Subsequently, phylogenetic trees constructed using two methods (UPGMA and neighbor-joining methods) also revealed the genetic relationships among the CTQ group, the CTT group, and 26 other reference populations. The various color modules in the trees represented the populations from different geographic regions. Supplementary Figure S2A presented a circular rooted phylogenetic tree built by the UPGMA method based on the *F*_ST_ values of the pairwise populations at the same 59 au-DIPs in the novel panel. The 28 populations involved were divided into two primary branches, one of which was dominated by seven populations from the African region and another included the other 19 populations from East Asia, South Asia, America, and Europe regions, as well as the CTQ and CTT groups. The CTQ and CTT groups formed a sister clade with the other five populations from East Asia. The basically analogous phylogenetic branch distributions also were observed in the radiation tree, which was established based on the allelic frequency data applying the Neighbor-Joining method (Supplementary Figure S2B).

### 3.5 Principal Component Analyses

**FIGURE 3:**
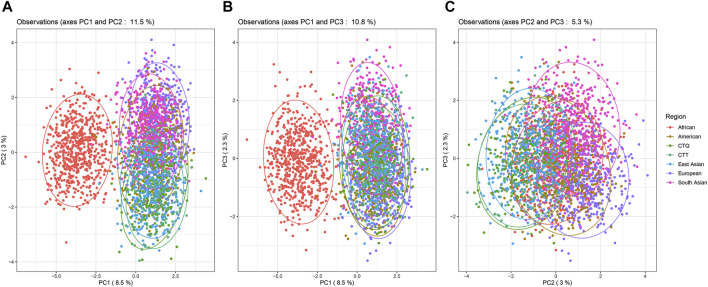
PCA results on the individual level among 2813 individuals from the CTQ and CTT groups, and other five different geographic region populations. **(A)** PCA results based on PC1 and PC2. **(B)** PCA results based on PC1 and PC3. **(C)** PCA results based on PC2 and PC3.

The PCA plots based on the population level (Supplementary Figure S3) and individual level (Figure 3) were conducted using the allele frequency data and allele genotyping raw data, respectively. In Supplementary Figure S3, the top three principal components (PCs) could explain 72.9% of genetic variance. PC1 (46.8%) and PC2 (16.1%) dispersed the European, African, and East Asian populations into three domains which were isolated from each other (Supplementary Figure S3A). PC1 (48.8%) and PC3 (10%) could separate the African and South Asian populations from other intercontinental populations (Supplementary Figure 3B). PC2 (16.1%) and PC3 (10%) mainly distinguished the South Asian populations from other intercontinental populations (Supplementary Figure S3C). In general, populations from the same geographic region clustered closer together, and the CTQ and CTT groups gathered with other populations from East Asia all the time. The contribution qualities for PC1 and PC2 of the 59 au-DIPs in the PCA plot were shown in a correlation circle (Supplementary Figure S3D), which was acquired with the square cosine values (Cos2, which is calculated as the squared coordinates), representing the contribution degrees to the PCs. The length from the center point to each variable represented the proportion of the variable in this dimension. In other words, the closer the locus to the circumference of the circle, the more important its contribution quality degree to PCA and the more effective it is in distinguishing these populations.

In Figure 3, the PCA distribution plots contained all individuals (sample size = 2813) from the CTQ and CTT groups and the other five different geographical regions. The first three PCs interpreted 13.8% of the total variation. The PC1 (8.5%) and PC2 (3%) plots (Figure 3A), and PC1 and PC3 (2.3%) plots (Figure 3B) significantly separated the African individuals and the individuals from other geographic regions. The PC2 and PC3 plots (Figure 3C) could not distinguish individuals from all geographic regions. Individuals from the CTQ and CTT groups tended to cluster together with individuals from East Asia in these three PCA results.

### 3.6 Population Genetic Architectures

**FIGURE 4:**
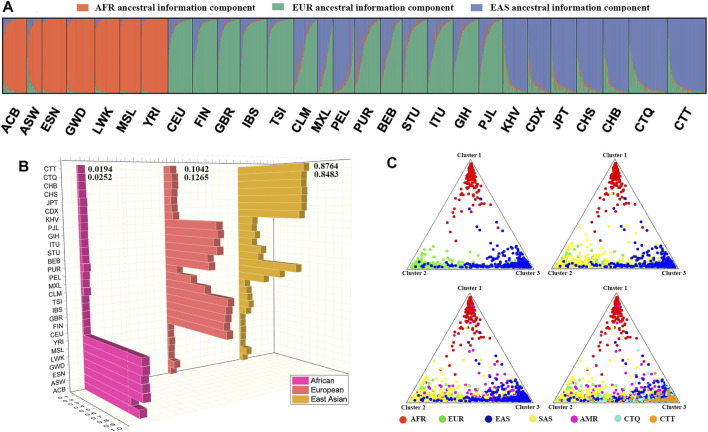
**(A)** Inferred population genetic structure by STRUCTURE analysis under a model with three ancestral components (*K* = 3). **(B)** Ancestral composition proportions of these 28 populations at *K* = 3; **(C)** triangle plots for individual ancestry estimations among the CTQ and CTT groups, and other five different geographic region populations. Dots with diverse colors represent individuals from different continental regions. EAS, East Asian; SAS, South Asian; AMR, American; EUR, European; AFR, African.

The STRUCTURE diagram via the Bayesian algorithm displayed the ancestral information components of all individuals. The predefined ancestral information components (*K* = 2–7) were distinguished by different colors (Supplementary Figure S4). The orange and green ancestral compositions were extracted at *K* = 2. The orange ancestral component was the African population, ranging from 98.39% (ESN) to 83.07% (African Ancestry in Southwest US, ASW) in the African seven populations. The predefined appropriate ancestral information component was three, which was determined by the online Structure Harvester program (Supplementary Figure S5). Herein, green, orange, and violet colors were defined as African, European, and East Asian ancestral compositions, respectively (Figure 4), and their proportions spanned from 97% (ESN) to 79.29% (ASW), from 89.47% (Toscani in Italy, TSI) to 84.01% (FIN), and from 87.64% (CTT) to 82.58% (CHB). The South Asian and American populations were considered mixed groups composing mainly of East Asian and European ancestral information components. The East Asian ancestral component proportion had the highest values in the CTQ and CTT groups, and the proportion values were 84.83% and 87.64%, respectively. The rest included the small amounts of European ancestral ingredient (12.65% in CTQ, 10.42% in CTT) and the negligible African ancestral composition (2.52% in CTQ, 1.94% in CTT). With the further addition of the *K* values, other ancestral compositions from South Asia and America regions manifested continuously. When defining five ancestral populations, these ancestral components corresponded to five different geographical regions one by one. The main ancestral component ratios of African, European, East Asian, South Asian populations ranged from 85.36% (ESN) to 66.53% (ASW), from 74.68% (TSI) to 65.87% (GBR), from 68.85% (CTT) to 54.67% (CDX), and from 68.92% (Sri Lankan Tamil in the United Kingdom, STU) to 59.4% (Bengali in Bangladesh, BEB), whereas the American ancestral composition had the largest value in Peruvian in Lima, Peru (PEL, 68.96%), followed by the Mexican Ancestry in Los Angeles, California (MXL); Colombian in Medellin, Colombia (CLM); and Puerto Rican in Puerto Rico (PUR) populations (46.65%, 30.52%, and 21.56%, respectively). At present, the East Asian ancestral component proportions in the CTQ and CTT groups were 61.98% and 68.85%, respectively (Supplementary Table S7). In the meantime, as shown in the triangle plot (Figure 4C), dots with diverse colors represented individuals from different intercontinental regions. These four triangle plots simulated the individual distributions from different geographic regions with the increase in the included populations. The first triangular plot included the African, European, and East Asian individuals, which were distributed in three different corners, representing the three main ancestral components. For the second triangle plot included African, European, East Asian, and South Asian populations, where South Asian individuals (yellow dots) were mainly distributed in the green and blue corners. Similarly, the AMR individuals were shown in the third triangle plot. The fourth triangle plot contained the African, European, East Asian, South Asian, and American individuals, as well as individuals from the CTQ and CTT groups. The results signified that the CTQ and CTT individuals were marked with turquoise and orange dots, respectively, and overlapped principally with the East Asian individuals, which were labeled with dark blue dots.

### 3.7 Mining Ancestry Informative Markers

#### 3.7.1 Population-Specific Divergence

The PSD values of these 59 au-DIPs among the five geographical populations, Africa, Europe, East Asia, South Asia, and America (represented by the PEL population), were summarized in Supplementary Table S8. The accumulated PSD value of the 59 au-DIPs in distinguishing the populations from the five geographical regions was 3.0359. The maximum PSD was found at rs35173752 (PSD = 0.1855), rs10579944 (PSD = 0.1526), and then rs5833522 (PSD = 0.1509). The cumulative PSD values of these loci in African, European, American, South Asian, and East Asian populations were 2.7545, 1.0462, 1.3185, 0.6476, and 0.7029, respectively. These loci could assign 98.49% African individuals, 86.08% European individuals, 92.94%, American individuals, 79.55% South Asian individuals, and 90.08% East Asian individuals to the intercontinental regions where they originated from (Supplementary Table S9). The closer the individual was to the corner, the more accurately it could be assigned to its original geographic region (Supplementary Figure S6).

#### 3.7.2 Informativeness for Assignment and *F*-Statistics

**FIGURE 5:**
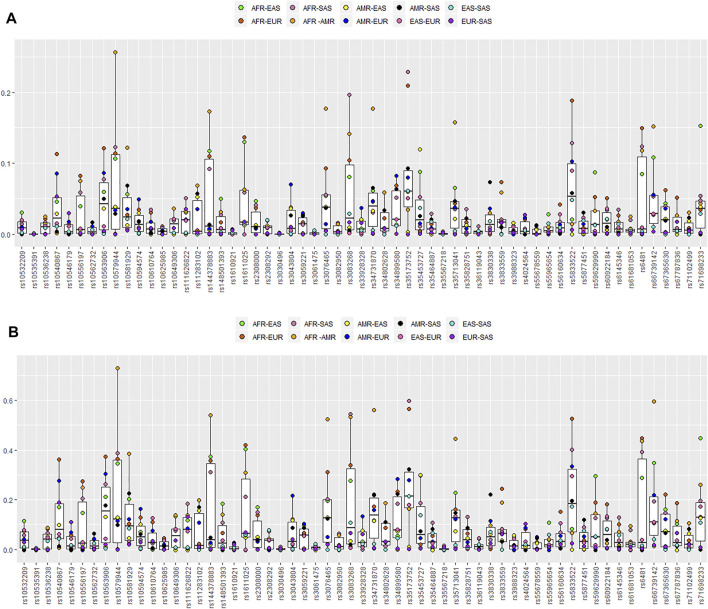
Box plots of pairwise *I*_n_ and locus-by-locus of *F*_ST_ values. **(A)** Pairwise *I*_n_ values for the 59 au-DIPs among the five different geographical region populations. **(B)** Pairwise *F*_ST_ values for the 59 au-DIPs among the five different geographical region populations.

The *I*_*n*_ values and locus-by-locus of *F*_ST_ values of these 59 au-DIPs for pairs of regional populations are summarized in Supplementary Table S10 and Figure 5. Herein, the threshold proposed for being a high-efficiency ancestry informative marker was the *I*_n_ value >0.131 (Rosenberg et al., 2003). The largest *I*_n_ value (*I*_n_ = 0.2563) was found in the African and American combination at the rs10579944 locus with the *F*_ST_ value of 0.7298. The rs144378883 (*I*_n_ = 0.1732), rs3076465 (*I*_n_ = 0.1767), rs3083268 (*I*_n_ = 0.1415), rs34731870 (*I*_n_ = 0.1770), rs35713041 (*I*_n_ = 0.1579), and rs66739142 (*I*_n_ = 0.1517) loci also showed higher *I*_n_ values (>0.131) with higher *F*_ST_ values of 0.5389, 0.5248, 0.5336, 0.5617, 0.4462, and 0.5957 between the African and American populations, respectively. In the same way, higher *I*_n_ values and *F*_ST_ values were also found at the rs71698233 (*I*_n_ = 0.1530, *F*_ST_ = 0.4481) locus between the African and East Asian populations and the rs1611025 (*I*_n_ = 0.1366, *F*_ST_ = 0.4189), rs35173752 (*I*_n_ = 0.2092, *F*_ST_ = 0.5650), rs5833522(*I*_n_ = 0.1822, *F*_ST_ = 0.5261), and rs6481 (*I*_n_ = 0.1495, *F*_ST_ = 0.4484) loci between the African and European populations and the rs3083268 (*I*_n_ = 0.1968, *F*_ST_ = 0.5454) and rs35173752 (*I*_n_ = 0.2283, *F*_ST_ = 0.5984) loci between the African and South Asian populations, respectively. These loci were thus considered as the promising marker to differentiate African and non-African populations.

## 4 Discussion

### 4.1 Forensic Application Efficacy Evaluation of the Novel System

The genetic polymorphisms of these DIPs included in this new homemade panel and its forensic efficiency for personal identification and paternity testing should be validated in relevant populations. So far, these forensic parameters have not been evaluated in Tibetan groups. Here, 155 CTQ and 154 CTT samples were genotyped by this new panel, respectively. The LD analysis and HWE exact testing illustrated that all pairwise loci were independent of each other, and the samples in this survey were representative. The PD and PIC values of the autosomal loci ranged from 0.5126 to 0.9496, and 0.2885 to 0.8094; and 0.5351 to 0.9297, and 0.3006 to 0.8022 in the CTQ and CTT groups, respectively. The autosomal loci in this new panel were also reasonably informative (PIC >0.25) in the two Tibetan groups (Botstein et al., 1980). This new panel also provided substantially lower CPM (1.9253E-27 in CTQ, 1.5061E-26 in CTT) than those obtained from the previously developed systems incorporating 35 DIPs (5.5662E-15 in CTQ, 9.6907E-15 in CTT) (Liu et al., 2020), and including 50 DIPs (about E-19–E-20) (Wang et al., 2020), indicating that this new system was more efficient for individual identification in Tibetan groups. Additionally, the CPE values generated in this system were higher than 0.9999 (0.999997 in CTQ, 0.99999895 in CTT), which was an appreciable improvement compared with other existing DIP kits such as the kits containing 30 DIPs (Li et al., 2019), 35 DIPs (Liu et al., 2020), and 50 DIPs (Wang et al., 2020) with CPE values less than 0.9999.

### 4.2 Genetic Affinity Comparison and Population Structure Inquisition

A robust population genetic analysis is a prerequisite for understanding the genetic background of Chinese nationalities. To deeply determine the potential admixture level of the Tibetan groups and the genetic relationships with other reference populations, a comprehensive population genetic analysis was performed employing multitudinous bioinformatics analysis softwares, including STRAF, Arlequin, DISPAN program, Genepop, TBtools, PHYLIY, MEGA, Origin, STRUCTURE, *R*, CLUMPP, Distruct, Infocal, Snipper, and so on.

First, the allele frequency distributions of the CTQ and CTT groups were most similar to other populations in the East Asia region, and these seven East Asian populations aggregated together in the different subordinate branches of East Asia in the allele frequency heatmap. The main usage of genetic distance is to measure the genetic divergence among species or populations across the world, including the ancestral relationship or differentiation degree (Jakobsson, Edge, and Rosenberg 2013; Goswami and Dagla 2017). Different types of genetic distances have been developed, of which the *F*_ST_ value and *D*_A_ distance are still used to summarize genetic variations within and among populations. They have their own unique evolutionary and statistical properties, and the *F*_ST_ value is generally considered to be the population differentiation caused by the difference in genetic structure (Weir and Cockerham 1984; Jakobsson, Edge, and Rosenberg 2013), while the *D*_A_ distance is developed under the hypothesis that genetic differentiation originates from genetic drift and mutation event (Nei, Tajima, and Tateno 1983; Jin et al., 2019). In this study, genotyping data of the 59 au-DIPs were used to analyze the hereditary distances among these 28 populations. The obtained results were basically consistent with a previously published study (Liu et al., 2020) and demonstrated that the genetic relationship between the CTQ and CTT groups was the closest, and then the CTQ and CTT groups had relatively closer affinities with Han Chinese populations than other populations from East Asia. Unsurprising, given that linguistics (Lu et al., 2016) and archeological data (Yang et al., 2017) have already suggested that the Tibetan group and Han populations split from their shared ancestral population roughly 6,000–4,725 years ago, and increasing numbers of Han Chinese also began to settle down in Tibet or the northern part of the Tibetan Plateau (Qinghai Province) after the peaceful liberation of Tibet in 1951 (Moore et al., 2000). While the CTQ group had more genetic similarity with CHB than the CTT group, it was inevitably related to the special geographic location of Qinghai on the northeastern margin of the Tibetan Plateau, which is a vital geographical corridor for population migrations and admixtures (Liu et al., 2021), and the CTQ group might thus gain more hereditary effects from other lowland populations.

Whereafter, the tree model is usually applied to explore the biological evolution history in the bioinformatics field, and genetic affinities among populations are generally displayed through phylogenetic trees (Takezaki and Nei 2008). Here, the populations from the same geographical regions were concentrated in the same branch (except for the American populations) by the phylogenetic reconstructions with different tree methods. The genetic affinities of the CTQ and CTT groups were significantly close and gathered first. The CTQ and CTT groups and other East Asian populations belonged to different sub-branches in the East Asian branch. PCA is used to extract important information by reducing the multivariate data dimensionality to two or three principal components, which can be visualized graphically (Lever, Krzywinski, and Altman 2017). We also conducted PCAs at both the population and individual levels among the two Tibetan groups and other global reference populations, these PCA plots reflected that the CTQ and CTT groups always converged with the subpopulations or individuals from the East Asia region under the first three PCs. Additionally, the correlation circle of PCA contribution quality offered the thought for the follow-up on the ancestry informative marker excavation. STRUCTURE analysis is applied using the model-based Bayesian iterative estimation algorithm to infer the origins of individuals with unknown population characteristics (Porras-Hurtado et al., 2013). Here, STRUCTURE analysis was ultimately carried out to infer the detailed genetic structures of the CTQ and CTT groups, and the result that the two Tibetan groups had the most similar genetic structures to the East Asian populations was confirmed once again, which was consistent with previous findings based on the other 35 au-DIPs. Finally, the three commonly used indicators, PSD, *I*_n_, and locus-by-locus of *F*_ST_ values, were selected to determine the level of informativeness provided by these markers about distinguishing different continental populations. Results revealed that there were more loci that could differentiate between African populations and other non-African populations. These DIPs would be used as potential ancestral information markers to be further explored in future forensic application.

## 5 Conclusion

In this study, the forensic application efficacy of this new self-made six-color fluorescence multiplex PCR system was validated in the CTQ and CTT groups. The obtained results showed that the novel system could be applied well to the individual identification and paternity testing in two Tibetan groups, especially for forensic applications of degraded biological materials. Furthermore, combining the previous research achievements of these two Tibetan groups, the comprehensive overview for the genetic relatedness and population structure of the Chinese Tibetan groups was explored in-depth again, and these findings were of great significance to further reveal the genetic background and structure of Chinese Tibetan groups in different regions.

## Data Availability

The datasets for this article are not publicly available due to concerns regarding participant anonymity. Requests to access the datasets should be directed to the corresponding author.
