# Supplementary material for: Forensic Efficiency Estimation of a Homemade Six-Color Fluorescence Multiplex Panel and In-Depth Anatomy of the Population Genetic Architecture in Two Tibetan Groups
Source: Front Genet. 2022 May 27;13:880346. doi: 10.3389/fgene.2022.880346 (PMC9184685; doi:10.3389/fgene.2022.880346)
Supplement: Supplementary file 1 [file DataSheet1.zip › Supplementary Material/Supplementary Figure 1-6.docx]

Supplementary Material

## Supplementary Figures 1-6


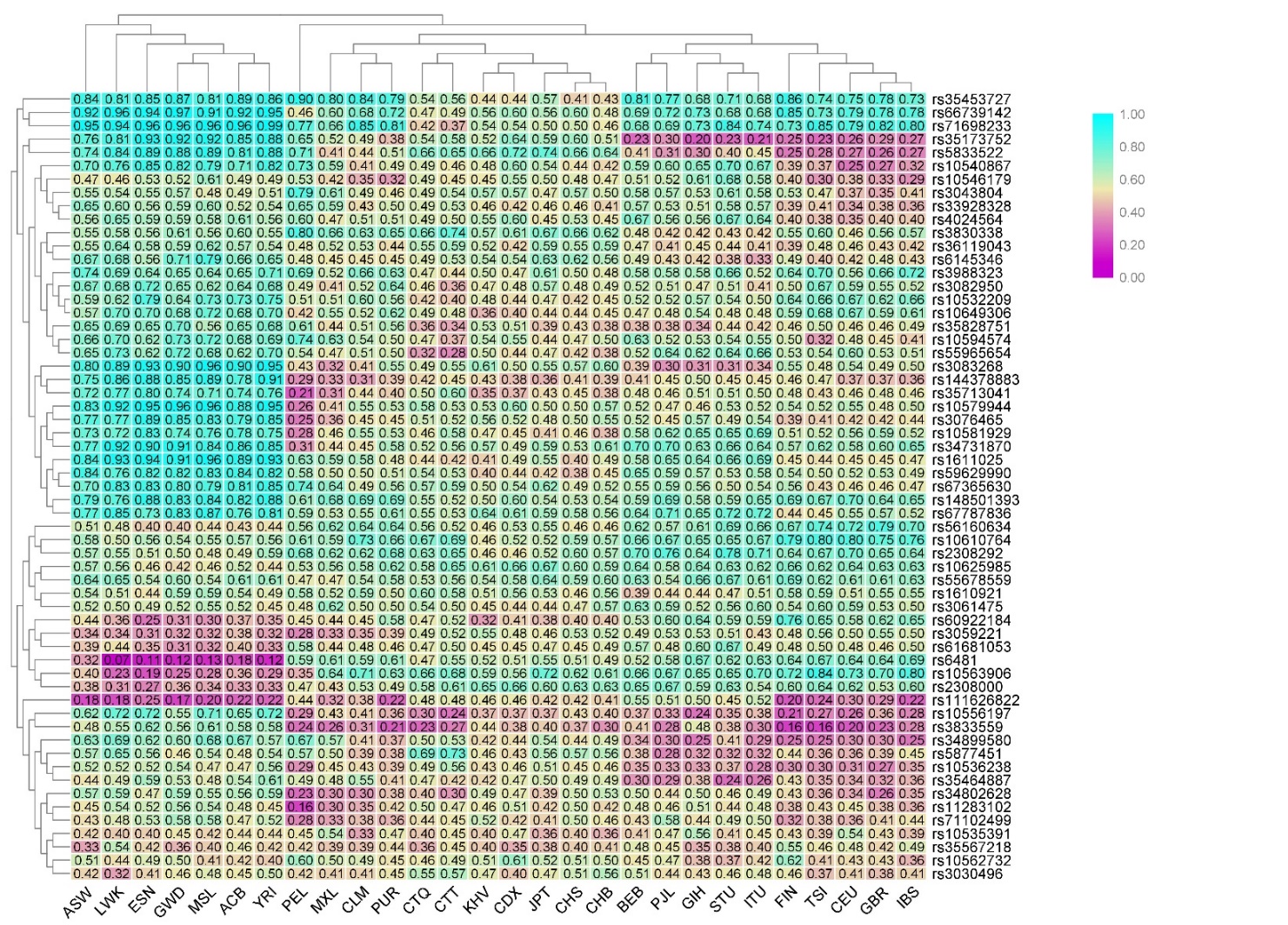


**Supplementary Figure 1 |** Cluster distribution heatmap of insertion allele frequencies at the 59 au-DIP loci in the 28 populations involved in this study. CTQ, Tibetan in Qinghai, China (n = 155) ; CTT, Tibetan in Tibet, China (n = 154); CDX, Chinese Dai in Xishuangbanna, China (n = 93); CHB, Han Chinese in Beijing, China (n = 103); CHS, Southern Han Chinese, China (n = 105); KHV, Kinh in Ho Chi Minh City, Vietnam (n = 99); JPT, Japanese in Tokyo, Japan (n = 104); PJL, Punjabi in Lahore, Pakistan (n = 96); GIH, Gujarati Indian in Houston, TX (n = 103); ITU, Indian Telugu in the UK (n = 102); STU, Sri Lankan Tamil in the UK (n = 102); BEB, Bengali in Bangladesh (n = 86); CLM, Colombian in Medellin, Colombia (n = 94); MXL, Mexican Ancestry in Los Angeles, California (n = 64); PEL, Peruvian in Lima, Peru (n = 85); PUR, Puerto Rican in Puerto Rico (n = 104); CEU, Utah residents with Northern and Western European ancestry (n = 99); FIN, Finnish in Finland (n = 99); GBR, British in England and Scotland (n = 91); IBS, Iberian populations in Spain (n = 107); TSI, Toscani in Italy (n = 107); ACB, African Caribbean in Barbados (n = 96); ASW, African Ancestry in Southwest US (n = 61); ESN, Esan in Nigeria (n = 99); GWD, Gambian in Western Division, The Gambia (n = 113); LWK, Luhya in Webuye, Kenya (n = 99); MSL, Mende in Sierra Leone (n = 85); YRI, Yoruba in Ibadan, Nigeria (n = 108).


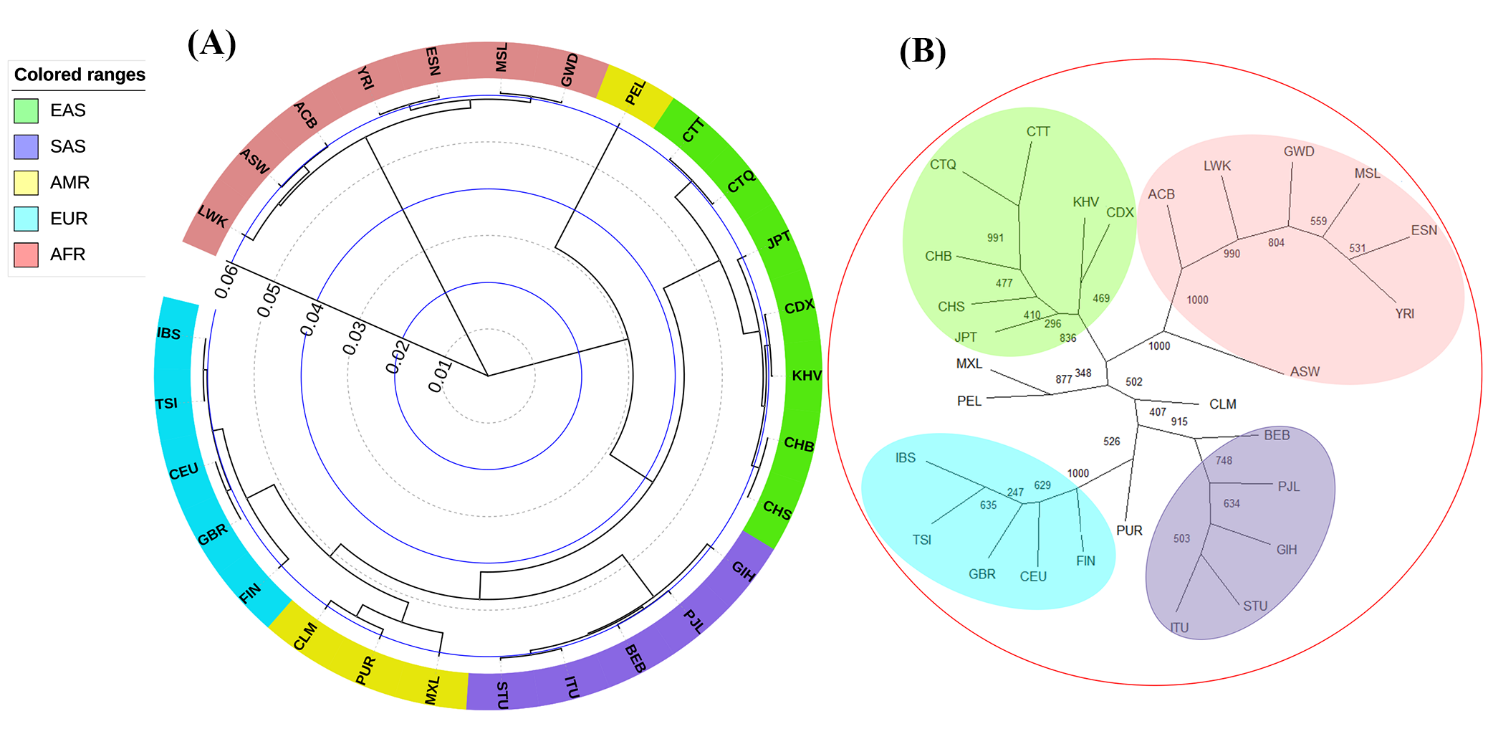


**Supplementary Figure 2 |** Two phylogenetic trees of CTQ, CTT groups and other 26 comparison populations. **(A)** Rooted evolutionary tree was built by UPGMA method based on the *F*_ST_ values among the pairwise populations at the 59 DIPs in the new panel; **(B)** Radiation evolutionary tree was constructed based on allele frequencies of the 59 DIPs in the new panel applying the Neighbor-Joining method. EAS, East Asian; SAS, South Asian; AMR, American; EUR, European; AFR, African.


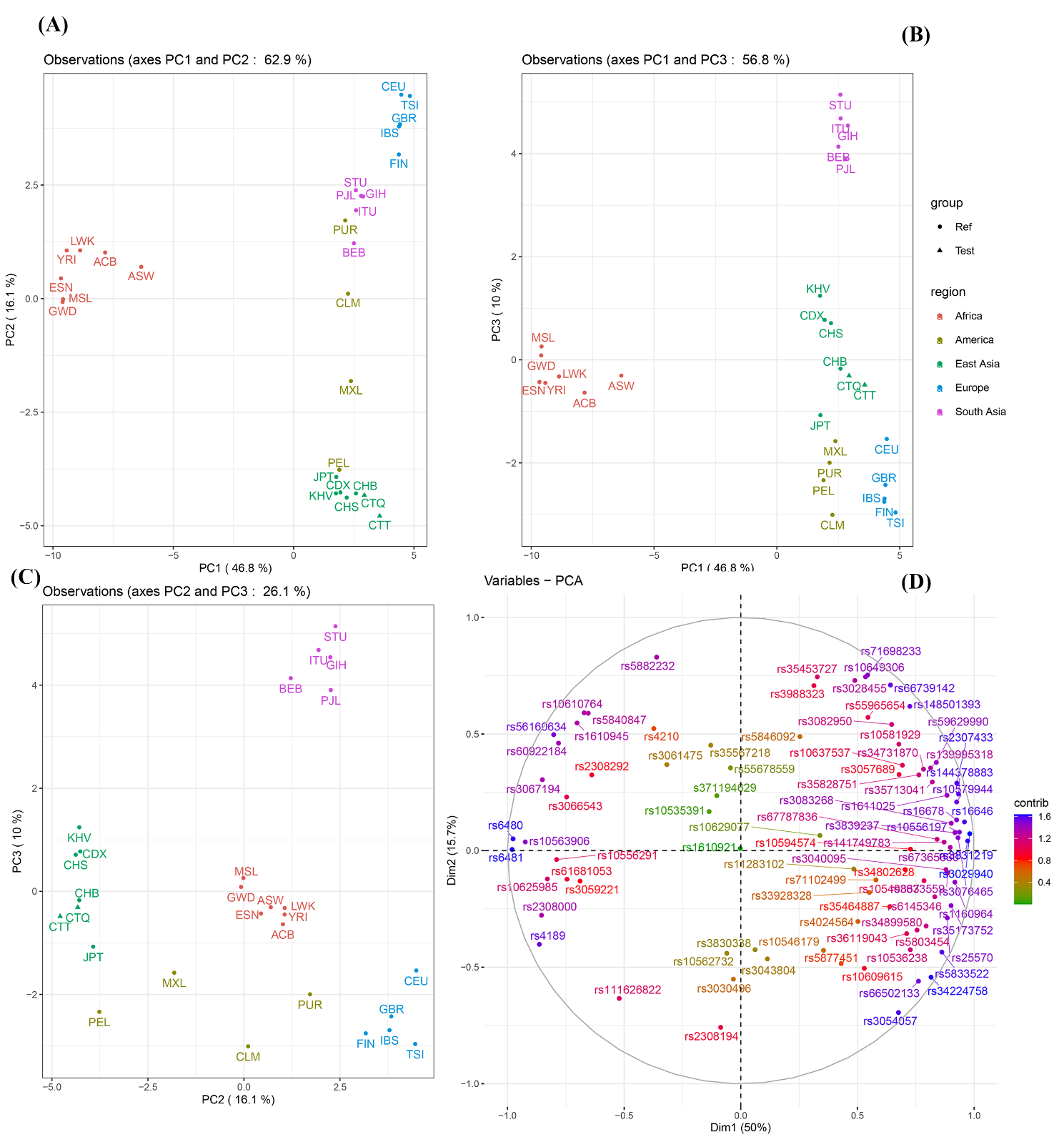


**Supplementary Figure 3 |** **(A)**, **(B)**, and **(C)** PCA results on population level based on the 28 populations. **(D)** The correlation circle for contribution qualities of 59 au-DIPs in PCA.


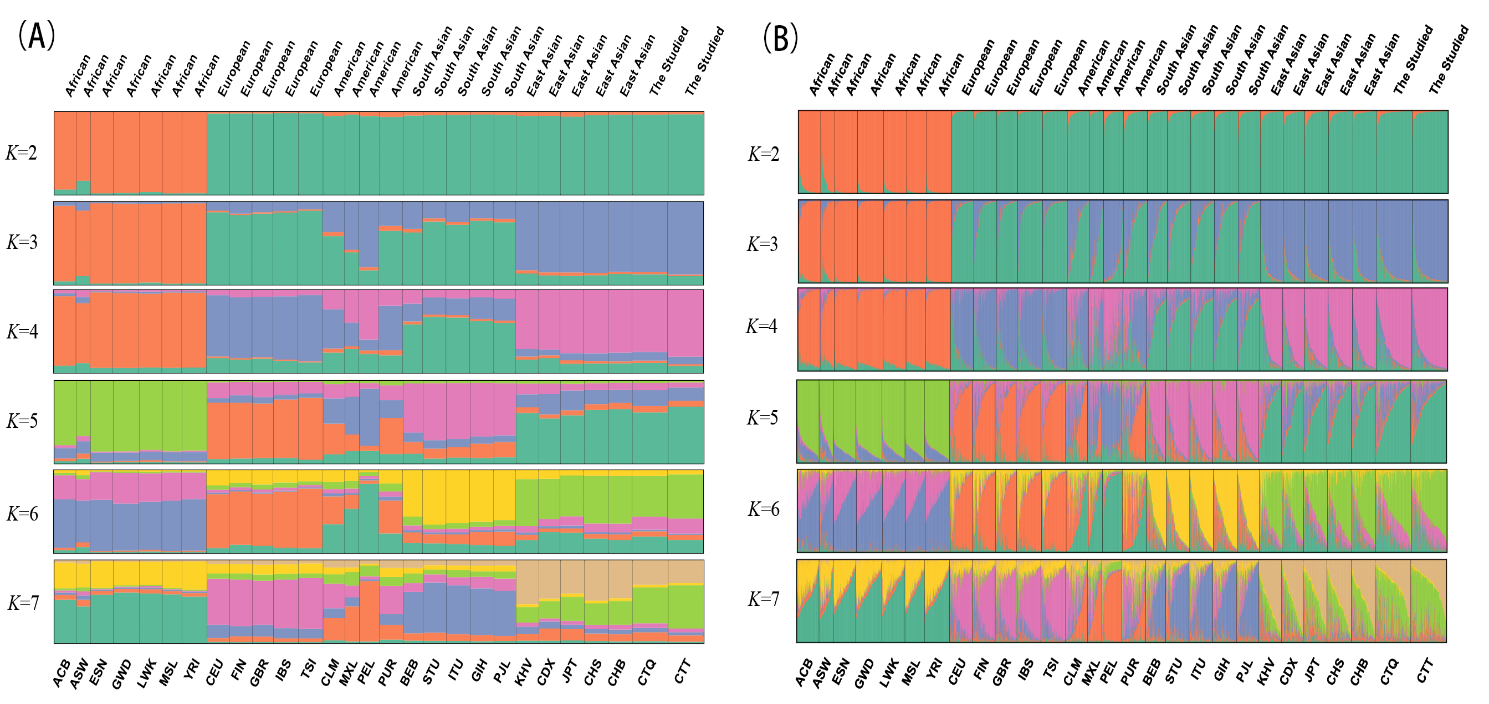


**Supplementary Figure** **4** **|** STRUCTURE analysis bar plots of 59 au-DIPs in the new panel (*K*=2-7). **(A)** the STRUCTURE analysis bar plots of 28 populations. **(B)** the STRUCTURE analysis bar plots of 2813 individuals from 28 populations. The different colors represented different ancestry components.


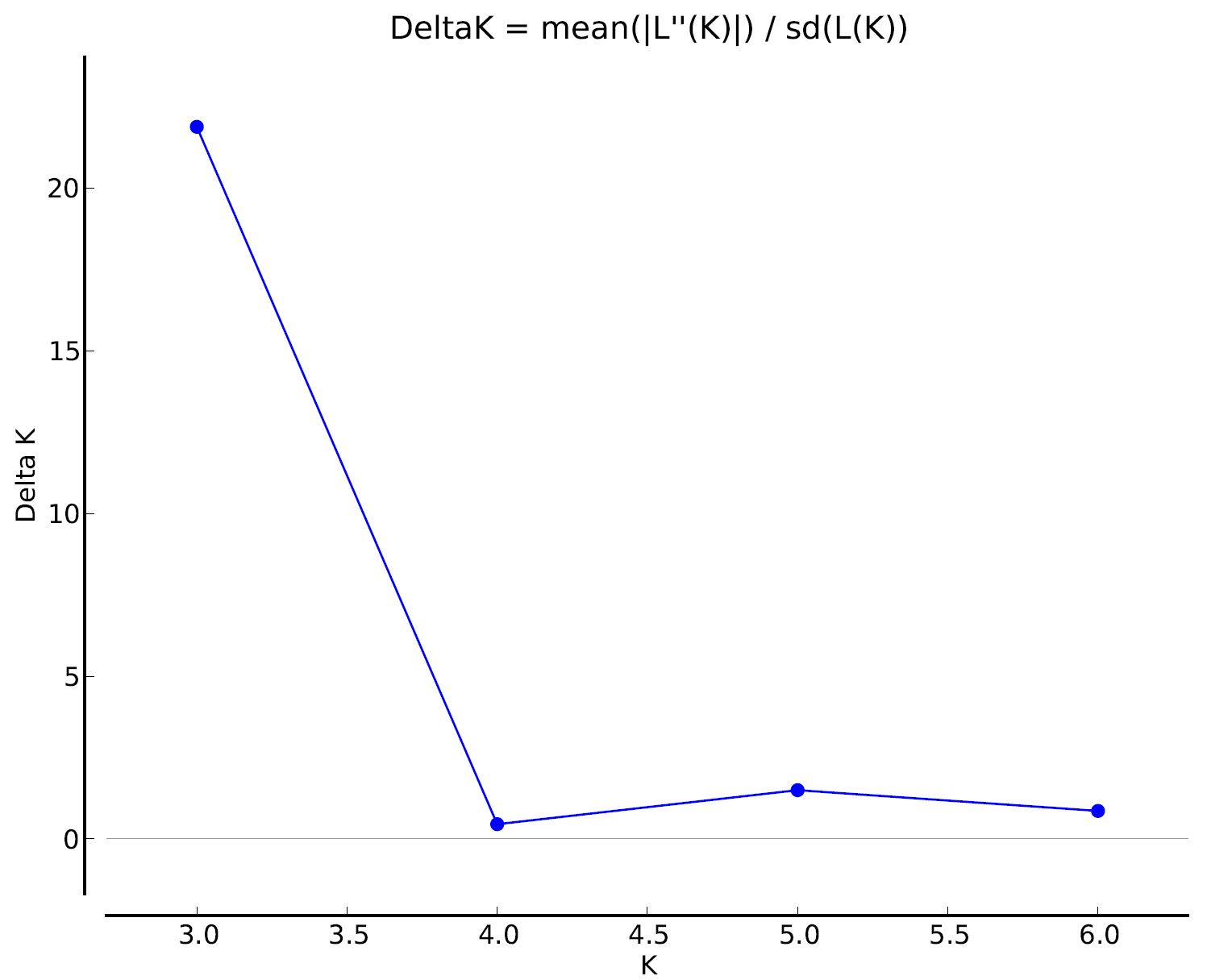


**Supplementary Figure 5 |** Structure Harvester program suggested that the optimum *K* value was 3.


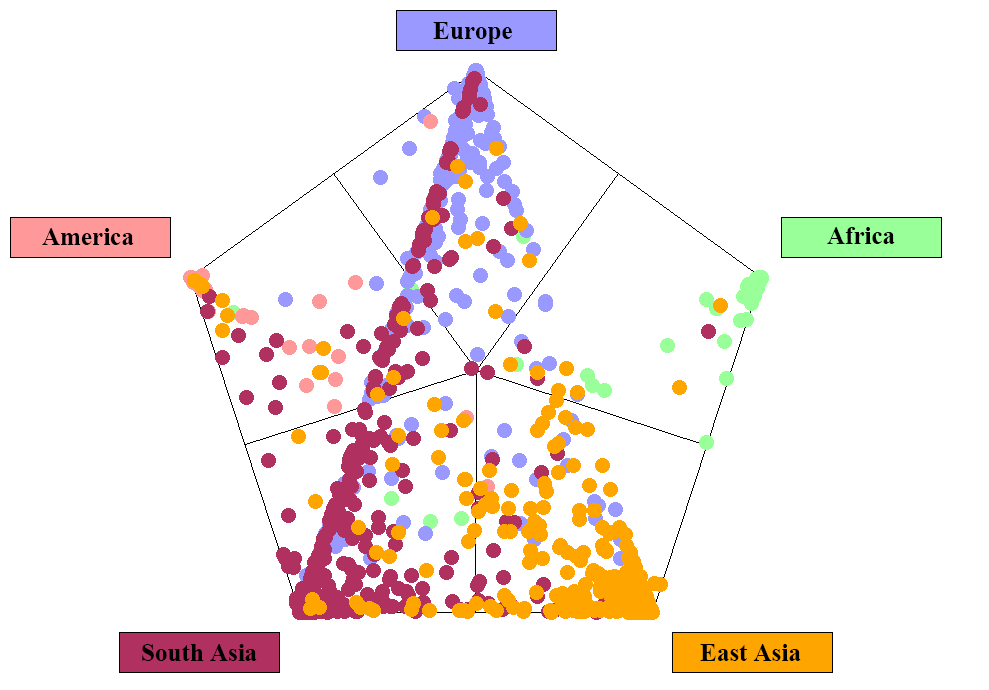


**Supplementary Figure 6 |** The individual classification results based on the PSD values of the 59 au-DIP loci in five geographical region populations.
